# Long-term benefit from high-dose ifosfamide in sarcoma depends on sustained prior control and timely intervention: a machine learning analysis

**DOI:** 10.1007/s00432-025-06410-8

**Published:** 2026-01-08

**Authors:** Michael Hoberger, Romy L. Zuber, Anton Burkhard-Meier, Dorit Di Gioia, Vindi Jurinovic, Michael Völkl, Sinan E. Güler, Markus Albertsmeier, Alexander Klein, Hans Roland Dürr, Nina-Sophie Schmidt-Hegemann, Thomas Knösel, Wolfgang G. Kunz, Michael von Bergwelt-Baildon, Lars H. Lindner, Luc M. Berclaz

**Affiliations:** 1 Department of Medicine III, University Hospital, LMU Munich, Marchioninistr. 15, 81377 Munich, Germany; 2 Bavarian Cancer Research Center (BZKF), Munich, Germany; 3 Institute for Medical Information Processing, Biometry, and Epidemiology, University Hospital, LMU Munich, Munich, Germany; 4 Department of General, Visceral and Transplantation Surgery, University Hospital, LMU Munich, Munich, Germany; 5 Orthopaedic Oncology, Department of Orthopaedics and Trauma Surgery, University Hospital, LMU Munich, Munich, Germany; 6 Department of Radiation Oncology, University Hospital, LMU Munich, Munich, Germany; 7 Institute of Pathology, LMU Munich, Munich, Germany; 8 Department of Radiology, University Hospital, LMU Munich, Munich, Germany; 9 German Cancer Consortium (DKTK), Partner Site Munich, Munich, Germany

**Keywords:** Bone sarcoma, Soft tissue sarcoma, High-dose ifosfamide, Machine learning-assisted modeling

## Abstract

**Purpose:**

High-dose ifosfamide (HD-IFO) remains an effective regimen for advanced bone and soft tissue sarcomas, but predictors of long-term benefit are poorly defined. This study evaluated clinical outcomes and prognostic factors using machine learning-assisted modeling in sarcoma patients treated with HD-IFO at a high-volume academic center.

**Methods:**

We retrospectively analyzed 26 patients with histologically confirmed bone or soft tissue sarcoma who received HD-IFO (≥ 12 g/m^2^ per cycle) between 2015 and 2025. Progression-free survival (PFS) and overall survival (OS) were estimated by the Kaplan–Meier method and compared across RECIST response categories using log-rank testing. Prognostic factors were identified using Least Absolute Shrinkage and Selection Operator (LASSO) logistic regression with leave-one-out cross-validation. The top three variables were entered into multivariable logistic regression to estimate odds ratios (ORs) for OS > 24 months.

**Results:**

Median PFS and OS from start of HD-IFO was 6.6 months (95% CI 4.4–9.8) and 24.7 months (95% CI, 14.7–34.2), respectively. Patients with progressive disease (PD) had significantly shorter OS than those with partial response (PR; *p* = 0.0047) or stable disease (SD; *p* = 0.0485). LASSO identified intervention prior to progression, prior tumor control ≥ 12 months, and absence of metastases as the strongest predictors for OS > 24 months. In multivariable analysis, intervention prior to progression (OR 24.18, 95% CI 1.81–1001.27, *p* = 0.037) and prior tumor control ≥ 12 months (OR 25.39, 95% CI 2.1–1008.9, *p* = 0.030) independently predicted OS > 24 months.

**Conclusion:**

HD-IFO provides durable disease control in selected sarcoma patients, particularly those with sustained prior tumor control and intervention prior to progression.

**Supplementary Information:**

The online version contains supplementary material available at 10.1007/s00432-025-06410-8.

## Introduction

Soft tissue and bone sarcomas are a heterogeneous group of malignant mesenchymal neoplasms characterized by diverse histopathological subtypes and clinical behaviors (Patrichi and Gurzu 2024). Despite advances in multimodal management, the prognosis of patients with advanced or relapsed sarcomas remains poor (Vos et al. 2019; Meazza and Scanagatta 2016).

High-dose Ifosfamide (HD-IFO) has long been recognized as an active cytotoxic agent against sarcoma, with a clear dose–response relationship and clinical activity even in previously treated patients (Le Cesne et al. 1995). Typically administered at cumulative doses ≥ 12–14 g/m^2^ per cycle, HD-IFO has demonstrated objective responses across multiple sarcoma subtypes, including synovial sarcoma, osteosarcoma, and well-differentiated/dedifferentiated liposarcoma (Patel et al. 1997; Sanfilippo et al. 2014). Early reports by Rosen et al. described an unusually high response rate in metastatic synovial sarcoma treated with HD-IFO, with all patients achieving measurable tumor regression (Rosen et al. 1994). Subsequent studies confirmed its efficacy in refractory or relapsed bone and soft tissue sarcomas, with objective response rates ranging from 20 to 40% and disease control rates exceeding 50% in selected subgroups (Palmerini et al. 2020; Lee et al. 2011; Martin-Liberal et al. 2013; Tirtei et al. 2024).

Continuous or prolonged infusion regimens have been introduced to improve the therapeutic index of HD-IFO, maintaining cytotoxic exposure while reducing neurotoxicity and myelosuppression. Sanfilippo et al. demonstrated that continuous 14-day infusional HD-IFO was feasible and effective, particularly in well-differentiated and dedifferentiated liposarcomas (Sanfilippo et al. 2014), while HD-IFO remained inactive in patients with myxoid liposarcoma (Colia et al. 2017). More recently, retrospective multicenter studies in relapsed osteosarcoma confirmed that outpatient HD-IFO protocols (14 g/m^2^ over 14 days) provide meaningful disease control with acceptable toxicity, supporting their integration into salvage therapy algorithms (Palmerini et al. 2020; Tirtei et al. 2024).

Long-term institutional publications on HD-IFO remain scarce, and predictive factors for response and survival are not well defined. In particular, the prognostic significance of clinical and histologic variables such as treatment line, correlation with previous response to ifosfamide, or best radiologic response, has not been systematically explored using modern analytic approaches. In this retrospective, single-institution study spanning over 10 years, we analyzed outcomes of patients with bone and soft tissue sarcomas treated with HD-IFO. We applied machine learning-assisted statistical modeling to identify prognostic factors associated with disease control and survival, focusing on overall survival (OS), and long-term disease control according to histological subtype and treatment response. This analysis aims to refine prognostic stratification and inform the optimal use of HD-IFO in the management of advanced sarcomas.

## Materials and methods

### Study design

An exploratory retrospective study design was chosen to evaluate clinical outcomes and prognostic factors in patients with bone and soft tissue sarcoma treated with HD-IFO at a high-volume academic center. All patients with histologically confirmed sarcoma and treated with HD-IFO between 2015 and 2025 were included.

### Patient selection

Eligible patients were adults (≥ 18 years) with histologically confirmed recurrent/metastatic soft tissue or bone sarcoma who had received at least one cycle of HD-IFO as part of their therapeutic management. Histopathological diagnoses were confirmed by an experienced sarcoma pathologist (TK). Clinical, pathological, and treatment-related data were extracted from the prospectively maintained LMU sarcoma database, and survival information was verified with the Bavarian Cancer Registry. Baseline characteristics included age, sex, Eastern Cooperative Oncology Group (ECOG) performance status, histologic subtype, *Fédération Nationale des Centres de Lutte Contre le Cancer* (FNCLCC) grade, number of prior local and systemic therapies, AJCC stage and metastatic status, and best radiologic response according to RECIST 1.1. Imaging was reviewed by an experienced sarcoma radiologist (WGK). If available, results from comprehensive genomic profiling were reported. Toxicity associated with HD-IFO treatment was systematically assessed.

### Treatment protocol

HD-IFO was administered at cumulative doses between 12 and 14 g/m^2^ per cycle, typically as continuous intravenous infusion over five to six days, combined with equimolar mesna (2-mercaptoethane sulphonate) uroprotection and standard antiemetic prophylaxis. Treatment cycles were repeated every 21–28 days depending on hematologic recovery and clinical tolerance. All patients received lipegfilgrastim subcutaneously 24–48 h after each chemotherapy cycle as primary prophylaxis against neutropenia.

### Statistical analysis

Progression-free survival (PFS), defined as the time from treatment initiation to progression or death, and OS, defined as the time from treatment initiation to death from any cause, were calculated using the Kaplan–Meier method. Differences between survival curves were assessed using the log-rank test. Because of the small sample size and the relatively large number of potential clinical predictors, reliable variable selection in this setting is inherently challenging. To address this limitation, a machine learning-based approach was employed to identify the most relevant variables associated with long-term survival (> 24 vs. < 24 months after initiation of HD-IFO). A Least Absolute Shrinkage and Selection Operator (LASSO) logistic regression was implemented to perform automated variable selection. LASSO applies a penalty term proportional to the absolute size of regression coefficients, shrinking less informative predictors toward zero and thereby retaining only the most relevant variables while discarding collinear or noisy features. Model performance was internally validated using Leave-One-Out Cross-Validation (LOOCV), training on n–1 patients and testing on the remaining one, repeated n times. The regularization strength (λ) was optimized by grid tuning across a logarithmic range (λ = 10⁻^4^–10⁰), and model quality was evaluated using the area under the receiver operating characteristic curve (ROC-AUC). Candidate predictors included demographic and clinical variables such as intervention after HD-IFO but prior to progression (yes/no), presence of metastases (yes/no), prior progression-free survival ≥ 12 months (yes/no), number of prior systemic therapies (1 vs. > 1), age group (> 40 vs. < 40 years), prior chemosensitivity (partial response to first or second-line therapy; yes/no), primary tumor location (extremity vs. other), sex (male/female), metastatic burden (none/oligometastatic vs. ≥ 5 lesions). and histological subtype (synovial sarcoma vs other). Analysis of the absolute regression coefficients was performed to evaluate variable importance from the fitted LASSO model in order to identify predictors most relevant for OS group separation. Due to the limited sample size, the subsequent exploratory multivariable logistic regression analysis was restricted to the three top-ranked predictors to estimate the direction and magnitude of their effects (odds ratios). All analyses were performed in R version 4.4.0 (R Foundation for Statistical Computing, Vienna, Austria) using the caret (v7.0.1), glmnet (v4.1.9), survminer (v0.5.0), and survival (v3.8.3) packages.

## Results

### Patient characteristics

The clinicopathologic characteristics of the patient cohort are summarized in Table 1. A total of 26 patients were included in the analysis (median age 35 years, range 21–72). Most patients had an ECOG performance status of 0 (61%) or 1 (31%) at treatment initiation. Fifty-eight percent were male. The predominant histological subtype was synovial sarcoma (84%), followed by osteosarcoma (8%), desmoplastic small round cell tumor (DSRCT, 4%), and malignant peripheral nerve sheath tumor (MPNST, 4%). Tumor grading according to FNCLCC was G2 in 42% and G3 in 46%. The primary tumor site was most frequently located in the extremities (46%), followed by the trunk (35%), retroperitoneum (15%), and head and neck region (4%). Comprehensive genomic profiling was available in 41% of patients. The most common molecular alterations (excluding canonical SS18::SSX fusions in synovial sarcoma) involved ATM (18%), ARID1A (9%), CTNNB1 (9%), PALB2 (9%), and NF1 (9%), while 46% of profiled cases showed no detectable alterations. At the beginning of HD-IFO treatment, 78% of patients presented with metastatic disease (AJCC stage IV), most commonly affecting the lungs (n = 17, 65%). One patient with AJCC stage I was included due to the irresectable tumor location in the pituitary stalk.

**Table 1:** Patient characteristics

Factor	Strata	n	%
Total		26	100
Age (years)	Median [Range]: 35 [21–72]		
ECOG at beginning of treatment	0	16	61
	1	8	31
	2	2	8
Sex	Male	15	58
	Female	11	42
Histological subtype	Synovial Sarcoma	22	84
	Osteosarcoma	2	8
	DSRCT	1	4
	MPNST	1	4
Grading (FNCLCC)	G2	11	42
	G3	12	46
	NA	3	12
Primary tumor location	Extremity	12	46
	Retroperitoneum	4	15
	Trunk	9	35
	Head/Neck	1	4
Comprehensive genomic profiling*	Yes	11	42
	No	15	58
Most common molecular alterations (canonical SS18::SSX fusions in synovial sarcoma excluded)	ATM Mutation	2	18
	ARID1A Mutation	1	9
	CTNNB1 Mutation	1	9
	PALB2 Deletion	1	9
	NF1 Mutation	1	9
	No alterations	5	46
AJCC stage at beginning of treatment	I	1	4
	II	4	15
	III	1	4
	IV	20	77
Presence of metastasis at beginning of treatment	Yes	20	78
	No	6	22
Organ sites of metastasis at beginning of treatment**	Lung	17	65
	Liver	3	12
	Abdominal	3	12
	Pericardial	1	4
	Nodal	2	8
	Mediastinum	1	4
	Bone	1	4
	Intrathecal	1	4

MPNST, Malignant peripheral nerve sheath tumor; DSRCT, Desmoplastic small round cell tumor

*Panel sequencing

**Multiple organ sites of metastasis can be listed per patient

### Treatment

Treatment modalities are summarized in Table 2. All 26 patients had received prior systemic therapy, most commonly doxorubicin/ifosfamide (42%) or doxorubicin/ifosfamide followed by trofosfamide maintenance (31%) as part of their first-line treatment. Overall, 77% of patients had been exposed to ifosfamide in previous chemotherapy lines before initiation of HD-IFO. Most patients had undergone previous surgical resection (96%) and radiotherapy (69%) of their primary tumor in an initially localized setting. The median number of systemic therapy lines before HD-IFO initiation was 1 (range, 1–5). The median number of HD-IFO cycles administered was 4 (range, 1–6). Four patients received a cumulative HD-IFO dose of 10 g/m^2^ during cycle 1 and were escalated from cycle 2 onwards. According to RECIST 1.1, partial response (PR) was achieved in 42%, stable disease (SD) in 38%, and progressive disease (PD) in 19% of patients. Dose reductions were required in 42% of cases, mainly due to hematologic toxicity (12%) or acute kidney injury (12%). Treatment was completed as planned in 69% of patients, while interruptions occurred due to toxicity (12%), patient refusal (12%), or disease progression (8%). After the end of HD-IFO, 58% of patients underwent a local or systemic intervention before disease progression, including surgery (9 patients) and systemic maintenance therapy with trofosfamide (n = 5) or pazopanib (n = 1).

**Table 2:** Treatment modalities

Factor	Strata	n	%
Total		26	100
Previous resection	Yes	25	96
	No	1	4
Previous radiotherapy	Yes	18	69
	No	8	31
Previous Systemic therapy	Yes	26	100
	No	0	0
Number of systemic therapy lines before initiation of HD-IFO	Median [Range]: 1 [1–5]		
Previous Ifosfamide use	Yes	20	77
	No	6	23
Previous Systemic therapy protocols*	Doxorubicin/Ifosfamide	11	42
	Doxorubicin/Ifosfamide followed by	8	31
	Trofosfamide maintenance		
	Pazopanib	3	12
	Trabectedin	3	12
	Mini-ICE	3	12
	Etoposide/ Doxorubicin/Ifosfamide	2	8
	Gemcitabine/Docetaxel	2	8
	Adriamycin/Cisplatin	1	4
	VIDE/VAC	1	4
	Doxorubicin monotherapy	2	8
	EURAMOS	2	8
	Carboplatin/Etoposide	1	4
	TIE	2	8
	Temozolomide/Irinotecan/Vincristin	1	4
	Cisplatin	1	4
Number of HD-IFO cycles	Median [Range]: 4 [1–6]		
Best radiologic response to HD-IFO (RECIST)	Partial response (PR)	11	42
	Stable disease (SD)	10	38
	Progressive disease (PD)	5	19
Dose Reduction	Yes	11	42
	No	15	58
Reason for dose reduction	Hematological toxicity	3	27
	Acute Kidney injury	3	27
	Neurotoxicity	2	18
	Other**	3	27
Reason for interruption	Treatment completed	18	69
	Disease progression	2	8
	Toxicity	3	12
	Patient refusal	3	12
Intervention prior to progression	Yes	15	58
	No	11	42
Type of intervention prior to progression	Surgery	9	60
	Systemic maintenance therapy	6	40

*Multiple systemic therapy lines can be listed per patient

**Other reasons for dose reduction include singular kidney after tumor resection (n = 1), interaction with paxlovid after SARS-CoV2 infection (n = 1), and Diamond Blackfan anemia in a patient with osteosarcoma (n = 1)

### Outcomes

**Fig. 1:**
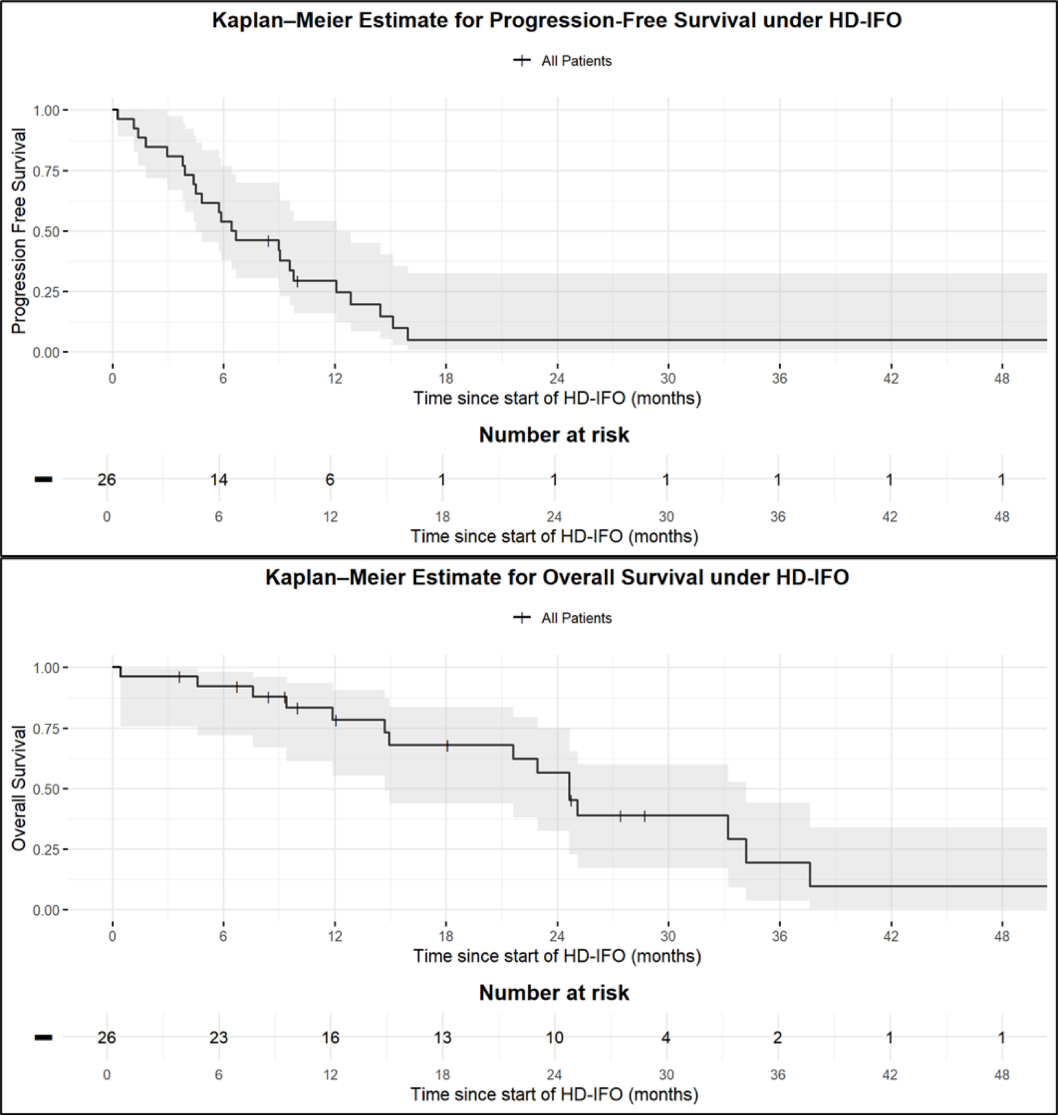
Kaplan–Meier estimate of PFS and OS under HD-IFO

The median PFS and OS from start HD-IFO were 6.6 months (95% CI, 4.4–9.8 months) and 24.7 months (95% CI 14.7–34.2 months), respectively (Fig. 1). Among patients with synovial sarcoma, the median PFS and OS from start of HD-IFO were 9.0 months (95% CI, 4.5–12.1 months) and 25.1 months (95% CI, 14.9–37.6 months), respectively (Supplementary Figs. 1–2).

**Fig. 2:**
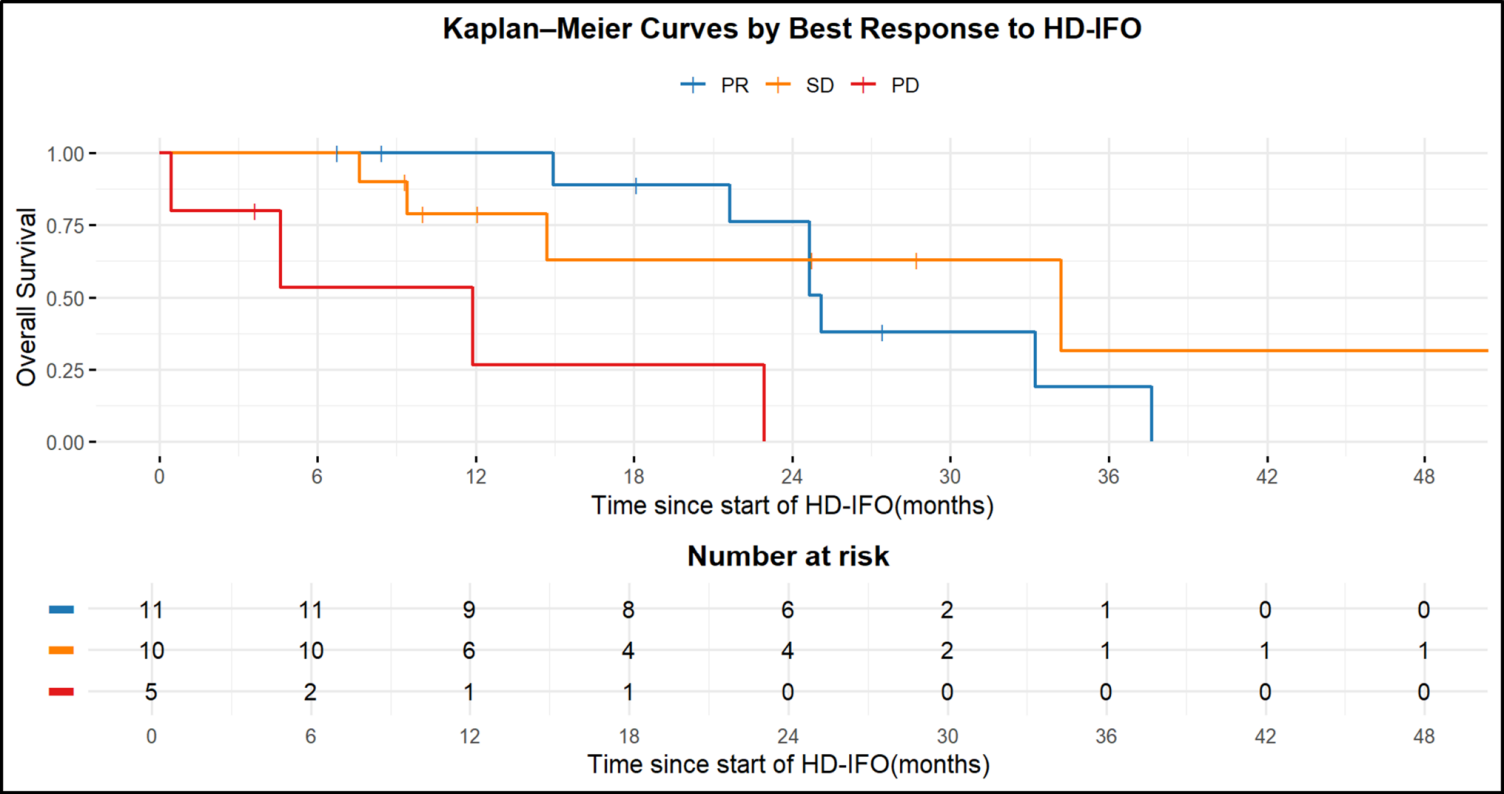
Kaplan–Meier curves of OS by best response to HD-IFO

Survival differed significantly among response groups (global log-rank test, *p* = 0.003). Pairwise comparisons showed that patients with PD according to RECIST had significantly shorter OS than those achieving PR (adjusted *p* = 0.0047) or SD (adjusted *p* = 0.0485), whereas OS did not differ between PR and SD (adjusted *p* = 0.5767) (Fig. 2). In the synovial sarcoma subgroup, the global log-rank test showed no significant difference in overall survival according to best radiological response to HD-IFO, with no separation between PR and SD. Interpretation of the PD group was not possible, as only a single patient had PD and this case was censored, preventing meaningful comparison with the other response categories. (Supplementary Fig. 3).

### Machine learning-guided OS modeling

Variable importance analysis from the LASSO logistic regression with LOOCV identified intervention (surgery, maintenance systemic therapy) prior to progression (relative importance = 100%), absence of metastases (relative importance = 66%), and prior tumor control ≥ 12 months (relative importance = 60%) as the most relevant predictors for long-term survival (> 24 months) following HD-IFO therapy. Other variables, including number of prior therapies (58%), age ≥ 40 years (52%), and prior chemosensitivity defined as PR to first- or second line chemotherapy (52%), showed moderate importance, whereas histological subtype and ECOG performance status contributed minimally. To determine the direction and magnitude of these associations, the three most relevant predictors were subsequently entered into a multivariable logistic regression. The model demonstrated an adequate fit (Null deviance = 31.49, residual deviance = 17.97). Both intervention prior to progression (β = 3.19, OR 24.18, 95% CI 1.81–1001.27, *p* = 0.037) and prior tumor control ≥ 12 months (β = 3.23, OR 25.39, 95% CI 2.1–1008.9, *p* = 0.030) were significantly associated with a higher likelihood of OS > 24 months. Absence of metastases was not significantly associated with OS > 24 months (β = 2.11, OR: 8.24, 95% CI 0.35–408.98, *p* = 0.21) (Table 3). Overall, the combination of LASSO-based variable selection and confirmatory logistic regression identified timely intervention before progression and durable prior disease control as key predictors of prolonged survival after HD-IFO treatment. In the synovial sarcoma subgroup, the reduced sample size required the use of Firth’s logistic regression to obtain stable effect estimates. Consistent with the findings from the overall cohort, intervention prior to progression was significantly associated with long-term survival (β = 2.43, OR 11.37, 95% CI 1.06–227.89, *p* = 0.044). Prior tumor control ≥ 12 months showed a strong trend toward improved OS (β = 2.10, OR 8.20, 95% CI 0.80–165.68, *p* = 0.077). In contrast, absence of metastases was not significantly associated with OS > 24 months (β = 1.57, OR 4.82, 95% CI 0.35–110.06, *p* = 0.24).

**Table 3:** Multivariable logistic regression analysis of predictors of prolonged OS (> 24 months) under HD-IFO therapy

Variable	OR	95% CI	*p*-value
Intervention prior to progression	24.18	1.81–1001.27	0.037
Prior tumor control ≥ 12 months	25.39	2.1–1008.9	0.030
Absence of metastases	8.24	0.35–408.98	0.213

OR, Odds ratio

## Discussion

HD-IFO remains one of the most active single agents for patients with advanced bone and soft tissue sarcomas in a further-line setting. In the present single-institution analysis, HD-IFO achieved a disease control rate of 81% (PR + SD) with a median PFS of 6.6 months and median OS of 24.7 months, outcomes that are superior to most historical series. Treatment was feasible, with dose reductions required in 42% of cases and discontinuation due to toxicity in only 12%, confirming that HD-IFO can be safely delivered even in pretreated patients when appropriate supportive measures are applied. We combined real-world clinical data from a decade-long institutional experience with machine learning-assisted modeling to derive a data-driven signature of long-term benefit from HD-IFO. By applying a LASSO-regularized logistic regression with LOOCV, we isolated intervention prior to progression and sustained prior tumor control ≥ 12 months as the most robust predictors of OS beyond 24 months.

Early work by Rosen et al. first demonstrated the potent antitumor activity of HD-IFO (14–18 g/m^2^ per cycle) in synovial sarcoma and long-term local control when used preoperatively (Rosen et al. 1994). Subsequent phase II studies by Le Cesne et al. and Patel et al. confirmed a clear dose–response relationship and the ability of HD-IFO to overcome resistance to standard-dose ifosfamide in advanced sarcomas (Le Cesne et al. 1995; Patel et al. 1997). Continuous-infusion regimens, introduced to mitigate neurotoxicity and renal complications, were later shown by Sanfilippo et al. to be both feasible and effective in well- and dedifferentiated liposarcoma, yielding durable disease stabilization in approximately half of patients (Sanfilippo et al. 2014). More recent retrospective series extended these observations to bone sarcomas. In a cohort of 51 osteosarcoma patients, Palmerini et al. reported an ORR of 20%, a 6-month PFS of 51%, and 2-year OS of 30% (Palmerini et al. 2020). Similarly, Tirtei et al., using a 14-day ambulatory infusion protocol, found a 4-month PFS of 54% and median OS of 13.7 months, again confirming meaningful disease control with acceptable toxicity (Tirtei et al. 2024). In this context, our data compare favorably (Table 4): the 42% PR rate observed in our cohort exceeds that of most prior series, and the median PFS of 6.6 months lies within the upper range of published reports (typically 3–6 months). The prolonged median OS of 24.7 months likely reflects both patient selection and the high proportion (62%) who underwent subsequent local or systemic intervention before progression, which are factors that our multivariable model identified as key predictors of long-term survival.

**Table 4:** Comparison of key response parameters to HD-IFO in bone and soft tissue sarcomas

Study	Number of patients	Histologic subtype	HD-IFO protocol	PFS	OS	Response (RECIST)
Hoberger et al. (2025)	26	Synovial sarcoma (81%), osteosarcoma (11%), DSRCT (4%), MPNST (4%)	12–14 g/m^2^ per cycle, continuous i.v. infusion over 5–6 days	mPFS 6,6 months	mOS 24,7 months	ORR 42% DCR 81%
Rosen et al. (1994)	13	Synovial sarcoma (100%)	14–18 g/m^2^ per cycle, continuous i.v. infusion over 6–8 days	mPFS ≈ 3 months (non-resected patients)	mOS not reached (median Follow-up 20 months)	ORR 100%
Le Cesne et al. (1995)	40	Leiomyosarcoma (30%), MPNST (17%), Fibrosarcoma (13%), Synovial Sarcoma (10%), UPS (7%), Liposarcoma (7%), Other (15%)	14 g/m^2^ per cycle, continuous i.v. infusion over 3 days	mPFS ≈ 8 months	mOS 12 months	ORR 33% DCR 55%
Patel et al. (1997)	74	STS (50%), BS (50%)	14 g/m^2^ per cycle, continuous i.v. infusion over 3 days	N/A	N/A	STS: ORR 19% BS: ORR 40%
Palumbo et al. (1997)	38	UPS (29%), Leiomyosarcoma (24%), Liposarcoma (18%), Synovial sarcoma (11%), Angiosarcoma (8%), Fibrosarcoma (5%), Rhabdomyosarcoma (5%)	14 g/m^2^ per cycle, continuous i.v. infusion over 4 days	mPFS ≈ 9 months	mOS 13 months	ORR 39% DCR 85%
Lee et al. (2011)	30	Leiomyosarcoma (23%), Synovial sarcoma (17%), Osteosarcoma (10%), Chondrosarcoma (7%), Ewing’s Sarcoma (3%), Others (40%)	12 g/m^2^ per cycle, continuous i.v. infusion over 6 days	mPFS 2,9 months	mOS 8,7 months	ORR 25% DCR 39%
Martin-Liberal et al. (2013)	35	DDLPS (62,8%) synovial sarcoma (20%), MRCLS (8,5%), others (8,5%)	14 g/m^2^ per cycle, continuous i.v. infusion over 14 days	mPFS 4,2 months	mOS 11,2 months	ORR 20% DCR 49%
Sanfilippo et al. (2014)	28	Liposarcoma (100%) (WDLPS 22%, DDLPS 78%)	14 g/m^2^ per cycle, continuous i.v. infusion over 14 days	mPFS 7,4 months	N/A	ORR 26% DCR 74%
Colia et al. (2017)	11	Myxoid liposarcoma (100%)	14 g/m^2^ per cycle, continuous i.v. infusion over 14 days	mPFS 1,9 months	mOS 37 months	DCR 14%
Palmerini et al. (2020)	51	Osteosarcoma (100%)	12–15 g/m^2^ per cycle, continuous i.v. infusion over 5 days	mPFS 6,1 months 6-month PFS 53%	mOS 14,5 months 2-year OS 52%	ORR 20%
Carter et al. (2020)	80 (46 STS, 34 BS)	STS: synovial sarcoma (33%), liposarcoma (28%), MPNST (7%), Others (32%) BS: Ewing sarcoma (47%), osteosarcoma 38%, Others (15%)	14 g/m^2^ per cycle, continuous i.v. infusion over 14 days	mPFS 3,4 months (STS: 3,8 months; BS: 2,5 months)	mOS 11,8 months (STS: 13,9 months; BS: 6,2 months)	STS: ORR 28% BS: ORR 17% DCR 41%
Tirtei et al. (2024)	26	Osteosarcoma (100%)	14 g/m^2^ per cycle, continuous i.v. infusion over 14 days	mPFS 4.1 months	mOS 13,7 months	ORR 23% DCR 57,7%

STS, Soft tissue sarcoma, BS, Bone sarcoma; WDLPS, Well-differentiated Liposarcoma, DDLPS, Dedifferentiated liposarcoma; MRCLS, Myxoid/round cell liposarcoma; MPNST, Malignant peripheral nerve sheath tumor; DSRCT, Desmoplastic small round cell tumor

As previously described, machine learning-assisted modeling in our study highlighted two independent predictors of prolonged OS: (1) intervention prior to progression (surgery or systemic maintenance therapy) and (2) durable prior tumor control ≥ 12 months. These findings align with the concept that systemic therapy serves as a bridge to local treatment. In the retrospective series by Palmerini et al., patients achieving a second complete surgical remission (CR2) after HD-IFO had the most favorable outcomes, suggesting that chemosensitivity sufficient to permit secondary surgery is a critical determinant of long-term survival rather than the cytotoxic effect alone (Palmerini et al. 2020). Our data extend this observation across histologic subtypes, including soft tissue sarcomas. Moreover, prior exposure to ifosfamide did not preclude benefit from HD-IFO in our analysis, consistent with prior evidence that dose escalation can overcome partial resistance mechanisms (Cesne et al. 1995). In contrast to the results reported by Palmerini et al. who described a significantly shorter PFS under HD-IFO in patients with recurrent or unresectable high-grade osteosarcoma previously treated with ifosfamide (Palmerini et al. 2020), our data did not confirm such an effect. This is consistent with the observations of Tirtei et al. (2024) and Carter et al. (2020), where no significant correlation was identified between prior ifosfamide exposure and treatment outcome. From our point of view, prior ifosfamide administration appears not to impact therapeutic efficacy and should not be considered a contraindication for re-treatment, particularly in the relapsed disease setting. Nevertheless, the magnitude of benefit appears attenuated in previously refractory patients, underscoring the need for rational patient selection based on prior chemosensitivity and interval since last progression.

The toxicity profile in our cohort mirrors published experience. Dose reductions due to hematologic or renal adverse events occurred in roughly 40% of cases, yet treatment completion was feasible in most patients. No treatment-related deaths were observed. Given the retrospective nature of our study, more detailed toxicity analyses were intentionally not pursued, and these data are reported primarily as pragmatic indicators of treatment feasibility rather than as a comprehensive toxicity assessment. Several studies have shown that ifosfamide demonstrates improved antitumor activity at doses exceeding 9 g/m^2^ per cycle in both soft tissue and bone sarcomas. These data highlight the importance of maintaining dose intensity to achieve optimal therapeutic benefit. Continuous infusion or protracted 14-day outpatient regimens may further improve tolerability without compromising efficacy and represent a promising approach for future studies (Palmerini et al. 2020; Tirtei et al. 2024). Lastly, ifosfamide-induced neurotoxicity (IIN) has been identified as an adverse prognostic factor in several studies, and patients who developed IIN showed significantly reduced PFS and OS compared with those without neurotoxic symptoms (Schmidt et al. 2022). In our cohort, the incidence of IIN prompting dose reductions was 18%, which lies at the lower end of the reported range, and was not significantly associated with reduced survival.

This analysis has several limitations. The study was based on a small and heterogeneous patient cohort, limiting statistical power and generalizability, as evidenced by the typically wide 95% confidence intervals observed in the logistic regression model. Notably, synovial sarcoma accounted for the majority of cases, while other histological subtypes were represented by very small numbers; therefore, the observed associations and outcome estimates are most robust for synovial sarcoma and should be interpreted with caution for other sarcoma entities. Its retrospective design entails potential selection bias and incomplete documentation, and only variables with sufficient data quality and variance could be included. We considered additional univariate analyses (e.g., for PFS/OS and individual prognostic factors such as treatment interruption, dose reduction, or prior therapy response). However, given the small sample size and the already exploratory nature of our analysis, performing multiple single-variable tests would substantially increase the risk of type I error and multiple-testing bias, and further univariate comparisons would likely yield statistically unstable and potentially misleading results. Although LASSO regression is appropriate for small datasets and supports built-in variable selection, it primarily identifies associations rather than causal relationships, and its measure of variable importance reflects the strength but not the direction of effects. The subsequent classical logistic regression used to estimate effect direction introduces additional methodological constraints, as both steps were performed on the same dataset, which can result in post-selection bias and overly optimistic effect estimates, the so-called “winner’s curse.” Accordingly, statistical inference from this model should be interpreted cautiously, and the findings should be considered exploratory and hypothesis-generating. Validation in larger, prospective, and independent cohorts is required to confirm these results and establish their clinical relevance.

## Conclusion

Our analysis reinforces the position of HD-IFO as an active and practical component of salvage therapy in advanced bone and soft tissue sarcomas. Machine learning-assisted modeling revealed that prolonged disease control before HD-IFO initiation and timely therapeutic intervention ahead of progression are decisive for achieving durable remission and extended survival. These insights provide a clinically actionable framework for refining patient selection and optimizing the integration of HD-IFO within multimodal sarcoma treatment strategies.

## Supplementary Information

Below is the link to the electronic supplementary material.

Supplementary Material 1

## Data Availability

The datasets generated during and/or analysed during the current study are available from the corresponding author on reasonable request.
