# Supplementary material for: Long-term benefit from high-dose ifosfamide in sarcoma depends on sustained prior control and timely intervention: a machine learning analysis
Source: J Cancer Res Clin Oncol. 2026 Jan 8;152(1):34. doi: 10.1007/s00432-025-06410-8 (PMC12783467; doi:10.1007/s00432-025-06410-8)
Supplement: Supplementary file 1 — Supplementary Material 1 [file 432_2025_6410_MOESM1_ESM.docx]

**Supplementary file, Hoberger et al. J. Cancer Res. Clin. Oncol. 2025**


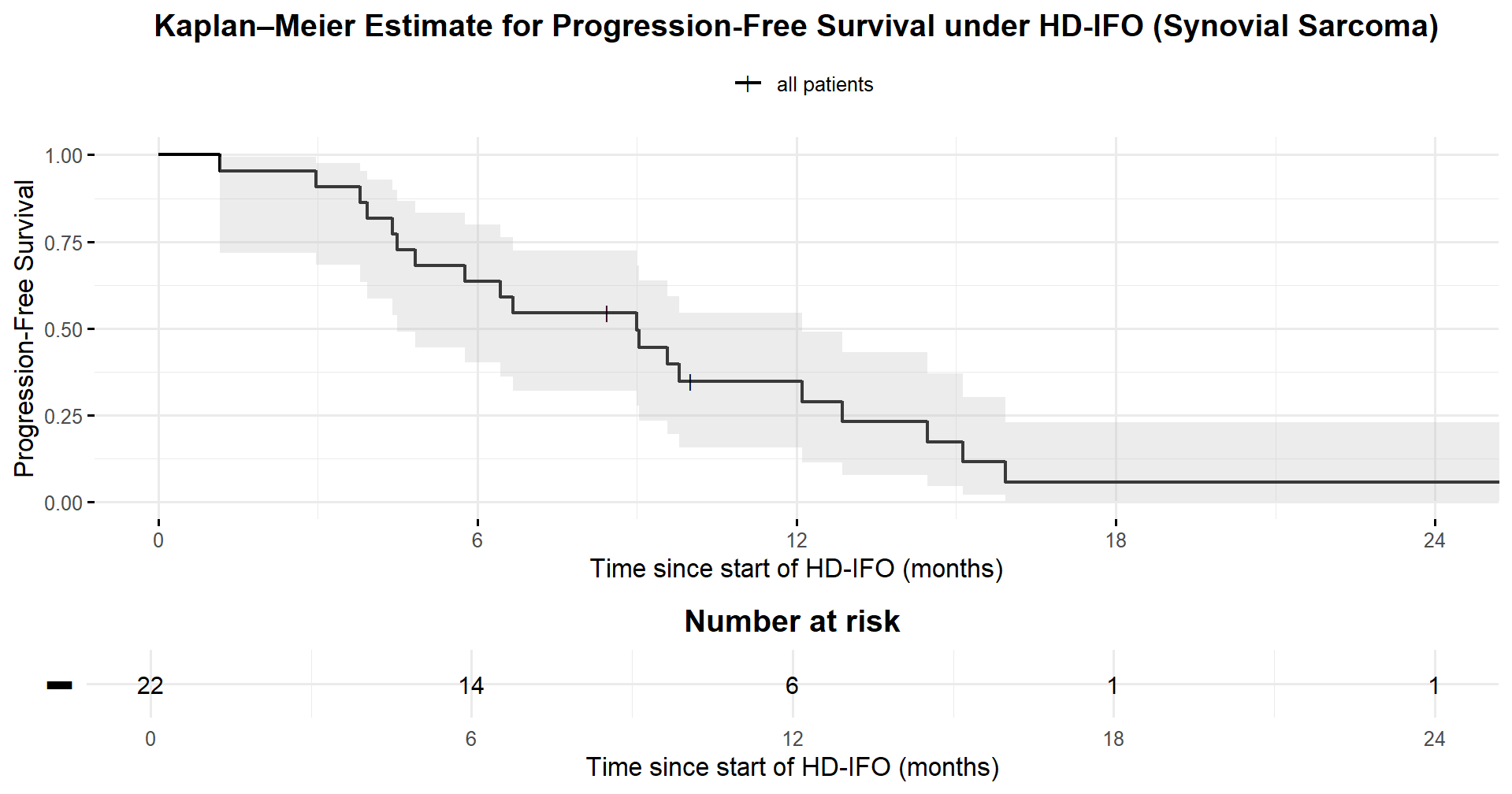


Supp. Figure 1: Kaplan-Meier estimate of PFS under HD-IFO in patients with synovial sarcoma.


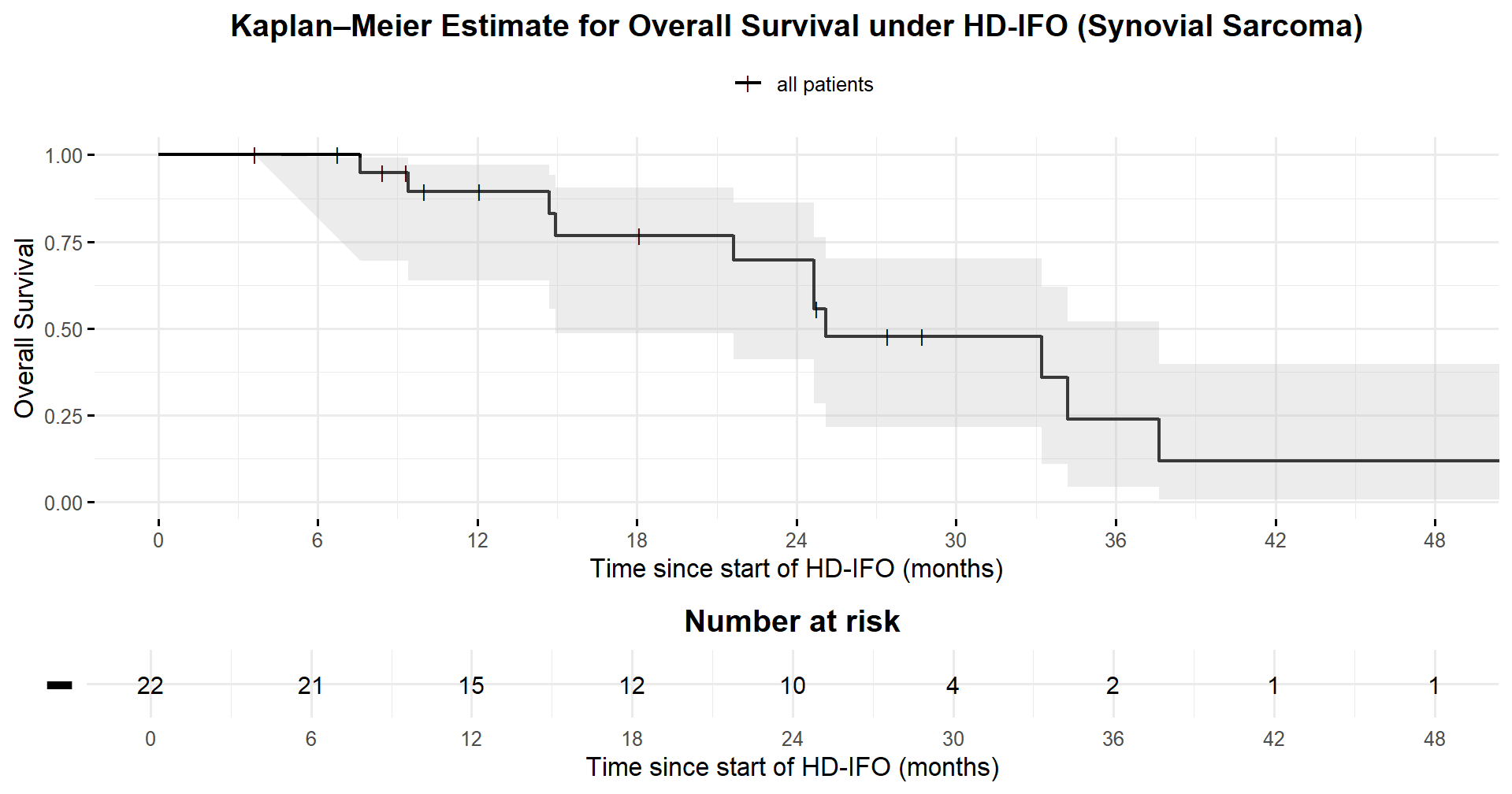


Supp. Figure 2: Kaplan-Meier estimate of OS under HD-IFO in patients with synovial sarcoma.


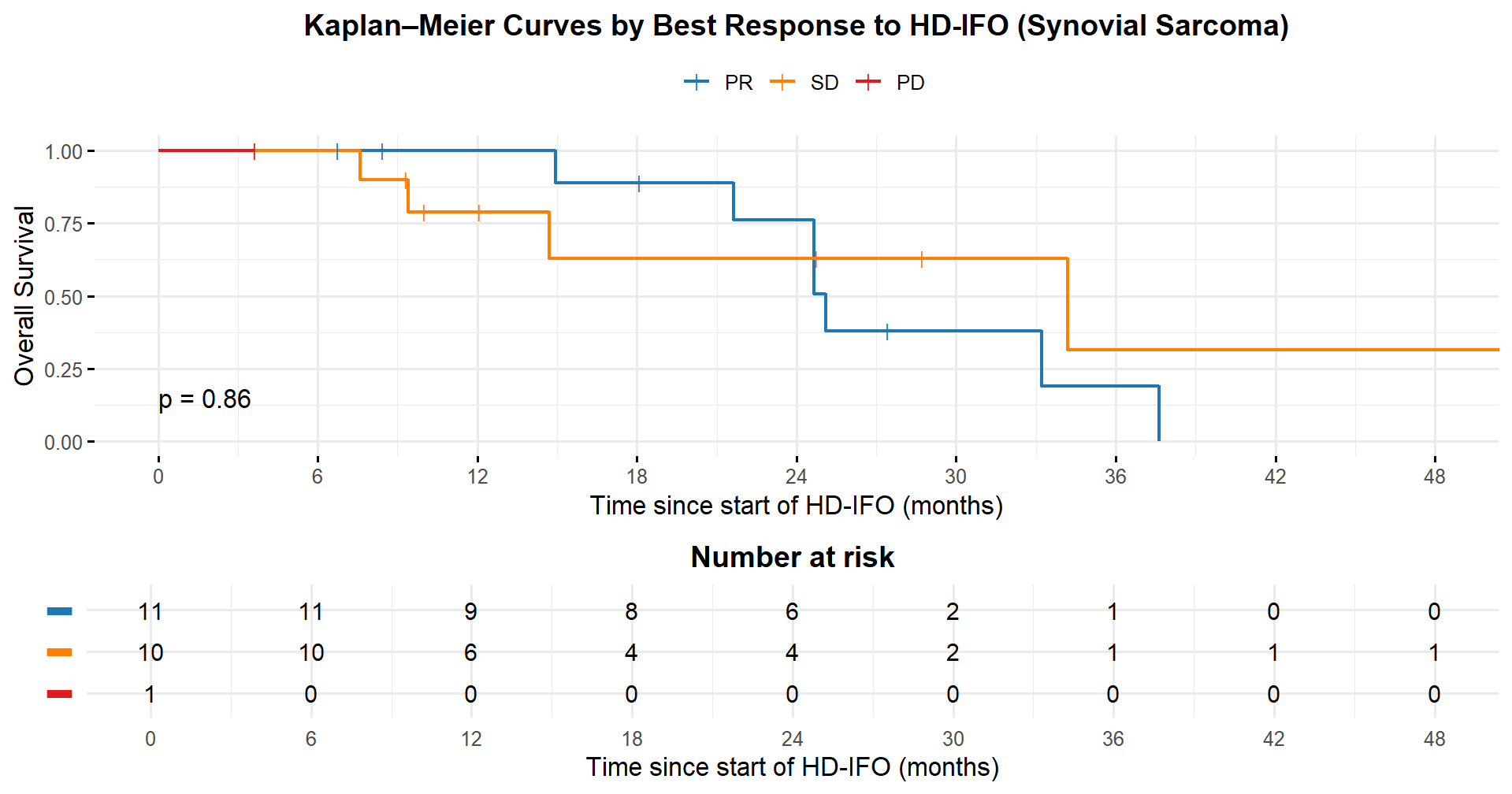


Supp. Figure 3: Kaplan-Meier curves of OS by best response to HD-IFO in patients with synovial sarcoma.
